# The Production Effect Interacts With Serial Positions: Further Evidence From a Between-Subjects Manipulation

**DOI:** 10.1027/1618-3169/a000540

**Published:** 2022-03-11

**Authors:** Sébastien Gionet, Dominic Guitard, Jean Saint-Aubin

**Affiliations:** ^1^ Ecole de Psychologie, Université de Moncton, Moncton, NB, Canada; ^2^ Department of Psychological Sciences, University of Missouri, Columbia, MI, USA

**Keywords:** production effect, between-participants design, free recall, serial positions, revised feature model

## Abstract

**Abstract:** Reading some words aloud during presentation, that is, producing them, and reading other words silently generate a large memory advantage for words that are produced. This robust within-list production effect is in contrast with the between-lists condition in which all words are read aloud or silently. In a between-lists condition, produced items are better recognized, but not better recalled. The lack of a between-lists production effect with recall tasks has often been presented as one of its defining characteristics and as a benchmark for evaluating models. Recently, Cyr et al. (2021) showed that this occurs because item production interacts with serial positions: Produced items are less well recalled on the first serial positions than silently read items, while the reverse pattern is observed for the recency portion of the curve. However, this pattern was observed with a repeated-measures design, and it may be a by-product of compensatory processes under the control of participants. Here, using a between-participants design, we observed the predicted interaction between production and serial positions. The results further support the Revised Feature Model (RFM) suggesting that produced items are encoded with more modality-dependent distinctive features, therefore benefiting recall. However, the production of the additional distinctive features would disrupt rehearsal.


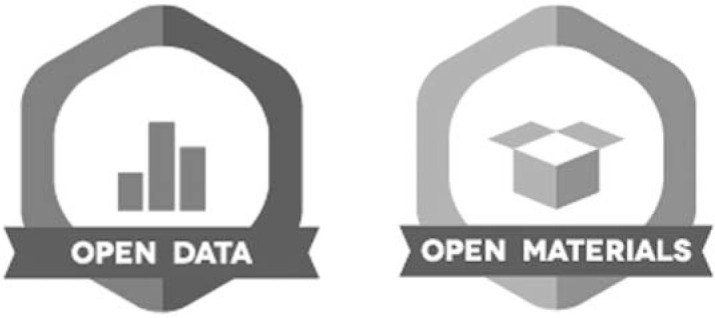


When some words within a list are read aloud and others silently, recall performance is systematically larger for words read aloud, that is, produced (see MacLeod & Bodner, 2017, for an overview). This production effect has been accounted for by calling upon distinctiveness processes; producing the items would make them more distinctive relative to silently read items (e.g., MacLeod et al., 2010). Accordingly, with a between-lists manipulation in which all items are produced or silently read, produced items would lose much of their relative distinctiveness advantage. With a recognition task, a smaller production effect has been found with a between-lists manipulation (Bodner et al., 2014; Fawcett, 2013), leading to the suggestion that a dual-process view could better fit the data than the single relative distinctiveness view. However, as predicted by the relative distinctiveness account, when memory is assessed with a free recall task instead of a recognition task, the between-lists production effect is systematically absent (see, e.g., Cyr et al., 2021; Forrin & MacLeod, 2016; Jones & Pyc, 2014; Lambert et al., 2016).

This asymmetry between recognition and free recall performance with pure lists has been identified by MacLeod and Bodner (2017) as an unresolved issue of major theoretical interest. Recently, Cyr et al. (2021) suggested that the lack of an overall production effect in free recall might be more apparent than real. When they assessed recall performance as a function of serial position, they uncovered an interaction with a large advantage of produced items on the recency portion of the curve and a large disadvantage on the primacy portion of the curve. They interpreted this finding with the Revised Feature Model (RFM) in which relative distinctiveness and rehearsal processes are key factors. However, as will be seen below, it is unclear if this interaction is a genuine effect or a by-product of participant-controlled strategies.

Serial positions have not been considered in studies of the production effect calling upon long-term memory tasks. This is anticipated in recognition studies in which serial positions are typically ignored. However, it is slightly uncommon in free recall. This leads us to question if serial positions have really been ignored in all previous free recall studies, apart from Cyr et al. (2021). This is important because before adapting theories to account for an effect, it is essential to demonstrate that it is reproducible in different laboratories (Oberauer et al., 2018). Therefore, we systematically reviewed the literature.

### Systematic Review of the Literature

In our systematic review of the literature, we considered all previous studies having measured the production effect with a free recall task as a function of serial positions. The literature search was conducted on August 19, 2021, on the PsycINFO and Scopus databases, and the following search terms were used **(**“Modality effect” OR “Vocalization effect” OR “Vocalization” OR “Vocalisation” OR “Production effect” OR “Reading aloud”) AND (“Free Recall” OR “Immediate Recall” OR “Serial Recall” OR “Verbal memory” OR “Serial learning”). Articles were considered for the review if they met the following inclusion criteria:

1) published empirical study,
2) used human participants,
3) compared memory performance for items being read aloud and read silently,
4) used a free recall memory task, and
5) included serial position curves or a table from which data could be extracted.

Studies including clinical populations were excluded from our review. In total, 462 studies were identified from database searching and from our previous work, and after removing duplicates, 338 studies were included in the primary screening.

The entire screening process was conducted by using the Covidence systematic review software (Veritas Health Innovation, Melbourne, Australia), and the flow diagram illustrating our search strategy and screening information is presented in Figure 1. For the primary screening, all studies were assessed by title and abstract and were then reviewed according to our inclusion and exclusion criteria. Of the 338 studies that were identified, 259 were excluded based on the title and abstract review, which yielded a total of 79 studies that were carried into the secondary screening phase for full-text review. After the remaining studies were assessed based on full-text, nine studies meeting all of our inclusion criteria were included in our review. Of the 70 studies that were excluded after the full-text review, 1 was a commentary, 1 could not be retrieved, 12 used a memory task other than free recall, 24 did not compare performance for items being read aloud and read silently, and 32 did not include a serial position curve or a table from which data could be extracted. Overall, we obtained 22 complete data sets coming from nine studies published from 1974 to 2021.

**Figure 1:**
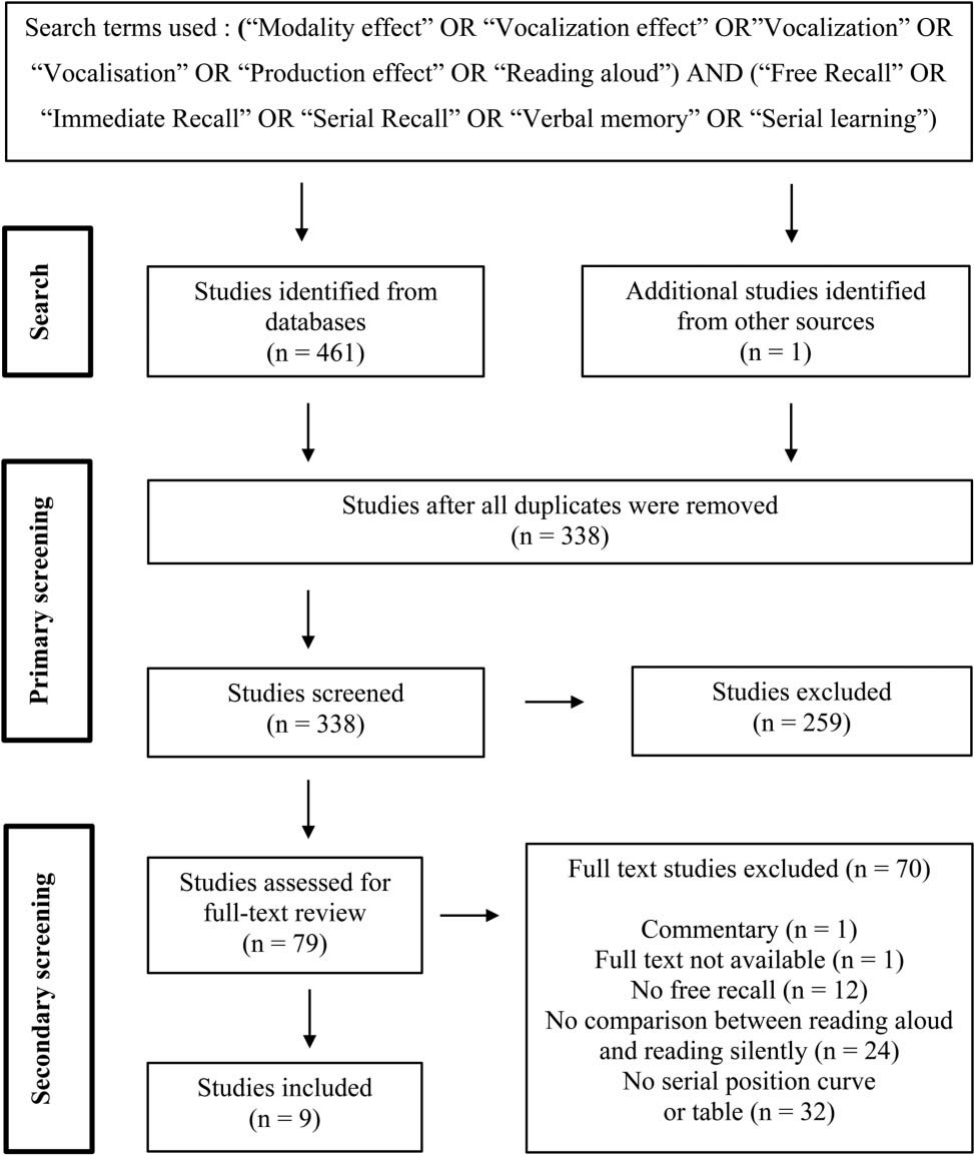
Flow diagram illustrating search strategy and screening information for the systematic review. Search terms used (“Modality effect” OR “Vocalization effect” OR “Vocalization” OR “Vocalisation” OR “Production effect” OR “Reading aloud”) AND (“Free Recall” OR “Immediate Recall” OR “Serial Recall” OR “Verbal memory” OR “Serial learning”).

After identifying our final sample of studies, we extracted data from each relevant experiment in which memory performance for items being read aloud and read silently was reported as a function of serial positions, and we then combined our extracted data into eight figures according to list length. When data were reported in a figure, we extracted the values by using the WebPlotDigitizer software, version 4.5 (Rohatgi, 2020). The list length for each study and experiment are displayed in Table 1, and the results from the data are summarized in Figure 2. Two main findings emerged from Figure 2. First, for all list lengths, produced items are better recalled for the recency portion of the serial position curve. Second, in most cases, silent items are better recalled than produced items on the primacy portion of the curve.

**Table 1:** List lengths used in the studies of the production effect as a function of serial position that were included in our systematic review

Source	Experiment	List length
Cyr et al. (2021)	1	8
	2	24
	3	10
Engle et al. (1989)	1	12
Engle and Roberts (1982)	1	12
Greene (1985)	5, 6, 7, 8, 9, 10	6
Greene and Crowder (1986)	2	6
Gregg and Gardiner (1984)	1, 2	6
Grenfell-Essam et al. (2017)	2	5, 6, 7, 8, 9, 12
Murray et al. (1974)	1	15
Watkins et al. (1974)	1	12

**Figure 2:**
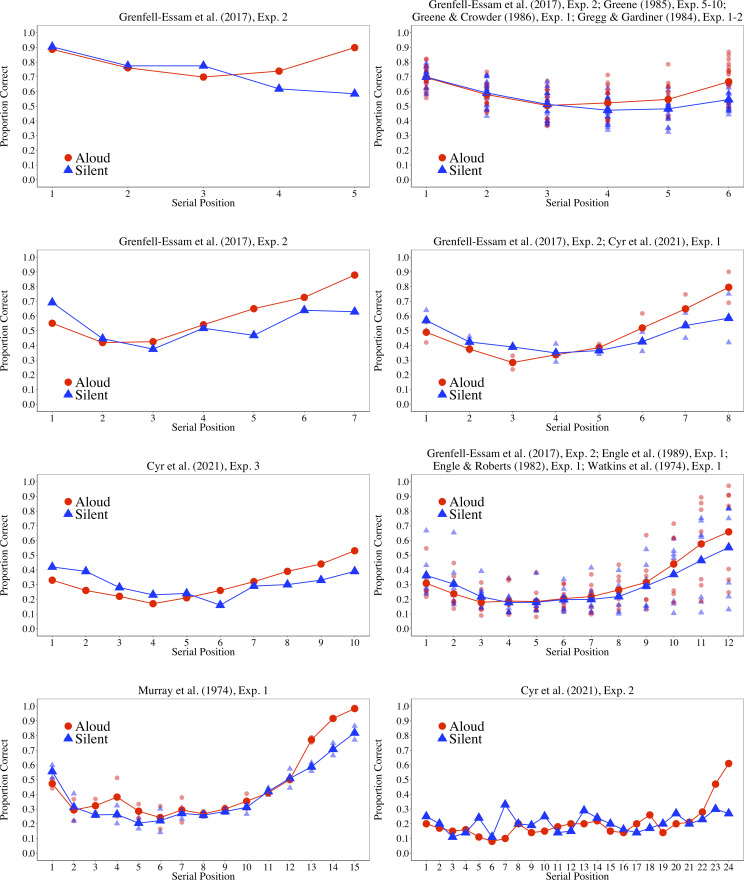
Average performance as a function of modality (aloud or silent) and serial position for each list length extracted from studies included in our systematic review. The transparent smaller circles and triangles represent average performance for each study.

### Current Study

The results of the systematic review are clear, but are they decisive enough to adjudicate models? One key methodological dimension must be mentioned. A within-participant design has been used in all studies included in our systematic review. This is in sharp contrast with the literature on the production effect in which separate groups are usually used to assess the between-lists production effect (MacLeod & Bodner, 2017). This methodological difference may prevent the generalization of the findings reported in the systematic review. With a repeated-measures design, participants are aware of all conditions and can assess their relative difficulty. This assessment may influence the choice of controlled processes, thereby influencing the observed pattern of performance, including the serial position curve in free recall (Unsworth et al., 2011). This is reminiscent of metamemory work showing that participants are likely to use the strategy they believe will be the most efficient to help them in a memory task (see, e.g., Koriat, 2000).

In a similar context, it has been argued that a within-participant design may elicit participant-controlled strategies (Watkins et al., 2000). More specifically, Watkins et al. investigated what they called the mixed-list paradox of the word frequency effect – the better recall of high- over low-frequency words. They suggested that when aware of the relative difficulty of two conditions, participants can adopt a strategy of compensating for what they anticipate will be more difficult to recall. They convincingly showed how these participant-controlled strategies can account for the different effect of word frequency in pure and mixed lists (but see Morin et al., 2006). They concluded their article by acknowledging that the role of study strategy in generating differential patterns of results in between-participants and within-participant effects of other variables remains largely unexplored.

In the context of the production effect, it can be argued that the single relative distinctiveness process is correct and that the observed interaction with serial position is driven by compensatory processes under the control of participants. More specifically, when asked to recall lists of words that have been read aloud and lists of words that have been read silently, participants would notice the greater difficulty of recalling silently read items. This would lead them to select a different control process for each list type. It is well-known that some processes will have a greater impact on the recency portion of the curve (e.g., imagery) while others have a greater impact on the primacy portion of the curve (e.g., elaborative rehearsal; Galli et al., 2012; Parker, 1981). If such a strategy explanation can account for the observed interaction between the production effect and serial position, it will greatly reduce the theoretical importance of the effect. In fact, it would be difficult to argue that this phenomenon can inform memory models. In the context of the production effect, the presence of an interaction deriving from participant-controlled strategies would legitimate past decisions of ignoring serial positions and the only key result would be the lack of a production effect with pure lists, as predicted by a single relative distinctiveness account (e.g., MacLeod et al., 2010).

Contrary to the single relative distinctiveness account, for the RFM, the interaction between the production effect and serial position is a genuine effect driven by basic memory processes (Cyr et al., 2021; Saint-Aubin et al., 2021). Therefore, the interaction between the production effect and serial positions observed in the systematic review should be fully observed with a between-participants design preventing usage of participant-controlled processes based on an assessment of the relative difficulty of each condition.

According to the RFM, item presentation generates vectors comprising modality-dependent and modality-independent features. Compared to silently reading the to-be-remembered items, reading them aloud would generate additional modality-dependent features (Cyr et al., 2021; Saint-Aubin et al., 2021). These additional features would increase the relative distinctiveness of the produced items. However, producing the items would induce a cost; it would interfere with rehearsal. This is akin to articulatory suppression in which saying an irrelevant item aloud impedes rehearsal (see, e.g., Murray, 1967). Furthermore, it is well-known that the first list items benefit more from rehearsal than the last list items (e.g., Bhatarah et al., 2009; Rundus, 1971). Within the RFM, after each item presentation, there is an attempt to rehearse all presented items so far. The rehearsal process restores some of the features lost due to interference. Therefore, producing the items would induce a disadvantage on the first serial positions due to impeded rehearsal and an advantage on the last serial positions due to enhanced distinctiveness. According to the RFM, none of these processes are under the control of participants. Consequently, the research design should not influence the interaction between the production effect and serial position.

In the current experiment, we contrasted the two views by assessing the production effect for pure lists with a between-participants design. At encoding, half of the participants read the items silently and the other half read them aloud. We used 10-item lists with a filled delay of 30 seconds. This procedure was used to allow comparison with Experiment 3 of Cyr at al. (2021), who used a within-participant design in which conditions varied randomly from trial to trial, maximizing opportunities for compensating strategies. According to the single distinctiveness view, in a between-participants design preventing the use of compensating strategies, recall performance for produced and silently read items should be equivalent on all serial positions. However, according to the RFM, the interaction observed in the systematic review should be present with a between-participants design.

## Method

### Sample Size Calculation

To determine our sample size, we used G*Power 3.1.9.4 (Faul et al., 2007) and the results of Experiment 3 of Cyr et al. (2021). More specifically, we used the effect size for the critical interaction between presentation modality (aloud vs. silent) and serial position (1–10) with the free recall procedure (η^2^_*p*_ = .05). With that information, an a priori interaction between a repeated-measures (serial position) and a between-participants factor (production) was computed with α = .05, power of .95, and the default parameters were used for the correlation among the repeated measures and the nonsphericity correction. The results from the analysis revealed that a total of 24 participants (12 participants in each group) were needed for our design. However, we decided to be cautious, as the effect size from Cyr et al. (2021) was based on a fully repeated-measures design and not a mixed design as used in the current study. We therefore overpowered our design and calculated a sensitivity analysis. The results from our analysis revealed that a total of 50 participants (25 participants in each group) with α = .05, power of .95, and the default parameters would allow us to detect a small effect (Cohen’s *f* = 0.16).

### Participants

Fifty students (30 females, 20 males, *M*_age_ = 19.32, *SD* = 1.33) from Université de Moncton participated in this study for course credits or were entered in a draw of $100. Participants were randomly and evenly assigned to one of the two presentation modality groups (25 participants silent and 25 participants aloud). To be eligible to participate in this study, participants had to be native French speakers, aged between 18 and 30 years with normal or corrected to normal vision, and who had never taken part in a study of the production effect. This last criterion was assessed by searching through our database in which the experiments in which all participants took part are noted and by asking participants at the end of the experiment. One participant was removed and replaced for not following the instructions. All participants gave their free and informed consent before the study, which was approved by the research ethics board of Université de Moncton.

### Stimuli

The stimuli were 220 French words selected from the Lexique 3 database (New et al., 2004). The stimuli were monosyllabic words, between three and seven letters and between two and five phonemes, with a frequency ranging from 0 to 1,321.79 occurrences per million (*M* = 66.85, *SD* = 162.71). The word pool was used to create 22 lists of 10 words with minimal phonological or semantic similarity within a list (see Appendix). Two of the lists were selected to serve as practice trials, and the remaining 20 lists were used for experimental trials.

### Design

A mixed design was implemented with presentation modality (read silently vs. read aloud) as the between-participants factor and serial position (1–10) as the within-participant factor. The experiment included 20 experimental trials preceded by two practice trials. The same two practice and 20 experimental lists of words were used for both presentation modality groups (aloud and silent), and the order of the words within a specific list was fixed for all participants. However, the order of the lists was randomized for each participant.^1^

### Procedure

Participants were tested in a single session lasting approximately 45 minutes in a quiet room and sat about 60 cm from the computer monitor. The experiment was controlled with PsytoolKit (Stoet, 2010, 2017), and the stimuli were displayed in lowercase in black 20-point Times New Roman against a white background. The experiment was self-controlled by the participants. Accordingly, participants initiated each trial by pressing the space bar key of the keyboard. For both presentation modality groups (aloud and silent), immediately after initiating a trial, the 10 to-be-remembered words were sequentially presented at the center of the computer screen at a rate of one word every 2 seconds (2,000 ms on, 0 ms off). Participants in the reading aloud group were instructed to read all words aloud while they were presented and to try to memorize as many words as possible. Participants in the silent reading condition received the same instructions, except they were told to read all the words silently, without moving their lips or whispering the words. After the presentation of the last word, participants had to complete a parity judgment task for 30 seconds (see Cyr et al., 2021). In the parity judgment task, a series of single integers from 0 to 9 were displayed at the center of the screen one at a time. Participants had to identify if the digit was an even number by pressing on the “M” key or an odd number by pressing on the “Z” key of a QWERTY keyboard. Immediately after the parity judgment task, three question marks were displayed at the top of the screen and served as a recall cue. Participants were instructed to recall as many words as possible from the last presented list of 10 words without consideration for their order. Participants typed the words with the keyboard and had to press the enter key after each word to register their answer. Recalled words remained on the screen once typed, and when participants were done recalling the items, they were instructed to press the enter key to skip the remaining items and to move on to the next list. The experimenter was present throughout the session to ensure compliance with the instructions.

### Data Analysis

In this study, our inferences were guided by Bayes factor (*BF*) independent *t*-tests and ANOVA analyses, which were computed with the “BayesFactor” package in R and the default parameters (Version 0.9.12–4.2; see Morey & Rouder, 2018; Rouder et al., 2009, 2012). In our *BF* ANOVA, participants were included as a random factor, the main effects and the interaction were tested by omitting the effects sequentially from the full model (Participant + Presentation modality + Serial position + Presentation modality: Serial position), 100,000 iterations were used to estimate our *BF*s via Monte Carlo simulation, and the proportional error was inferior to 5% for all *BF*s. In the Results section, we used the following nomenclature in which *BF*_10_ represents evidence in favor of an effect and *BF*_01_ (1/*BF*_10_) represents evidence against an effect. For *BF* ANOVA, the benchmarks taken from Kass and Raftery (1995) were used to facilitate the interpretation of our results. In addition to the BF analysis, we reported the corresponding *F* ratios, partial eta squares, *t*-tests, and Cohen’s *d* as descriptive information computed with the “ez” package (Version 4.4–0; Lawrence, 2016) and “lsr” package (Version 0.5; Navarro, 2015).

Before any statistical analyses were conducted, participants’ responses were checked for misspelling. Participants’ responses that could be identified without ambiguity were corrected (e.g., letter omissions: uscle instead of muscle; letter repetition: muuscle instead of muscle; substitution: muzcle instead of muscle). In the Results section, we report analyses with spelling corrected, but the same pattern of results was observed with incorrect spelling albeit with a slightly lower overall performance. The data with or without spelling corrections are available on the Open Science Framework page (https://osf.io/32msj/).

## Results

Participants’ responses were considered correct if a word was recalled, independently of its recall position. The proportion of correct responses was then evaluated as a function of presentation modality (aloud and silent) and input serial position. We also evaluated performance in the parity judgment task. More specifically, like Cyr et al. (2021), we explored the number of parity judgment attempts and the proportion of correct attempts (number of correct attempts/number of attempts) as a function of presentation modality group.

### Parity Judgment

For the parity judgment task, overall, participants in the aloud group (*M* = 43.48, *SD* = 10.69) made slightly more parity judgment attempts than participants in the silent group (*M* = 39.41, *SD* = 6.70). However, the results from Bayesian and Welch’s independent *t*-tests revealed more evidence in favor of the absence of difference between the two groups, although only superficial evidence was found, *BF*_01_ = 1.23 (aloud group = silent group), *t*(40.33) = 1.61, Cohen’s *d* = 0.46. For the proportion of correct parity judgments, participants’ performance was similar between the silent group (*M* = 0.96, *SD* = 0.03) and the aloud group (*M* = 0.94, *SD* = 0.09), but again, only superficial evidence was found for the null hypothesis, *BF*_01_ = 2.25 (aloud group = silent group) *t*(28.69) = 1.05, Cohen’s *d* = 0.30. Overall, these results suggest that participants were engaged in the parity judgment task and performance was relatively comparable between the groups.

### Free Recall

**Figure 3:**
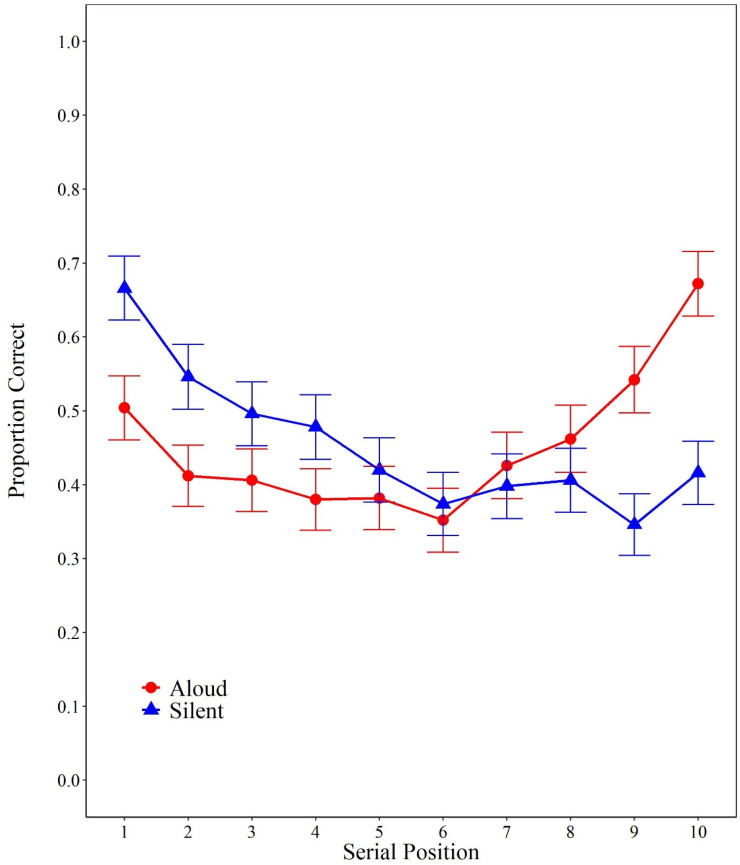
Proportion correct as a function of presentation modality group (aloud vs. silent) and input serial position (1–10). Error bars are 95% confidence intervals computed according to Morey’s (2008) procedure.

For free recall, overall, participants’ performance was nearly identical between the silent group (*M* = 0.455, *SD* = 0.15) and the aloud group (*M* = 0.454, *SD* = 0.14). However, as can be seen in Figure 3, and as expected, participants in the silent group were better for initial serial positions and participants in the aloud group were better for the last serial positions.

The results from the analyses of variance confirmed these trends. There was positive evidence against an effect of encoding condition, *F* < 1, η^2^_*p*_ = .00, *BF*_01_ = 9.20, and very strong evidence in favor of the main effect of serial position, *F*(9, 432) = 12.71, η^2^_*p*_ = .21, *BF*_10_ > 10,000. Most importantly, and as predicted, there was very strong evidence in favor of the interaction between encoding condition and serial position, *F*(9, 432) = 13.69, η^2^_*p*_ = .22, *BF*_10_ > 10,000. The latter critical interaction was further investigated by conducting independent Bayesian factor *t*-tests as a function of encoding group for each serial position. As shown in Table 2, performance between serial positions three and eight was relatively similar across presentation modality groups. Most importantly, participants in the silent group were better for Position 1 (*positive evidence*) and 2 (*superficial evidence*), while participants in the aloud group were much better than those in the silent group for Position 9 (*strong evidence*) and 10 (*very strong evidence*).

**Table 2:** Descriptive statistics (*M* and *SD* presented in parentheses) and independent Bayesian *t*-tests contrasting the two presentation modality groups (aloud vs. silent) for each serial position (1–10)

Position	Aloud	Silent	*BF*	Cohen’s *d*
Position 1	0.50 (0.23)	0.67 (0.19)	*BF*_10_ = 5.47	0.78
Position 2	0.41 (0.26)	0.55 (0.21)	*BF*_10_ = 1.50	0.58
Position 3	0.41 (0.22)	0.50 (0.20)	*BF*_01_ = 1.38	0.43
Position 4	0.38 (0.21)	0.48 (0.19)	*BF*_01_ = 1.07	0.49
Position 5	0.38 (0.19)	0.42 (0.19)	*BF*_01_ = 2.87	0.20
Position 6	0.35 (0.12)	0.37 (0.17)	*BF*_01_ = 3.15	0.15
Position 7	0.43 (0.15)	0.40 (0.17)	*BF*_01_ = 3.00	0.18
Position 8	0.46 (0.14)	0.41 (0.19)	*BF*_01_ = 1.96	0.34
Position 9	0.54 (0.18)	0.35 (0.20)	*BF*_10_ = 38.56	1.02
Position 10	0.67 (0.18)	0.42 (0.25)	*BF*_10_ = 194.95	1.19
*Note*. *BF*_10_ corresponds to evidence in favor of a difference between the aloud group and the silent group (aloud ≠ silent) while *BF*_01_ corresponds to evidence in favor of an absence of difference between the groups (aloud = silent).				

## Discussion

This study was aimed at testing the presence of an interaction between encoding conditions and serial positions with pure lists in which all items are either read aloud or read silently. The current results revealed the expected better recall of silently read items than of produced items on the first serial positions and the reverse pattern on the last serial positions. This interaction between the production effect and serial position nicely reproduced results previously observed with a within-participant design in which the same participants took part in the silent and the aloud conditions (Cyr et al., 2021; Engle et al., 1989; Engle & Roberts, 1982; Greene, 1985; Greene & Crowder, 1986; Gregg & Gardiner, 1984; Grenfell-Essam et al., 2017; Murray et al., 1974; Watkins et al., 1974). In this context, the comparison with the third experiment of Cyr et al. (2021; see Figure 2) is of particular interest. In effect, we used the same word pool, the same list length, and the same procedure as Cyr et al., except that we implemented a between-participants design with only a free recall task and a retention interval of 30 seconds instead of 2 minutes. A comparison of Figure 2 and Figure 3 reveals a very similar pattern of results between the two studies. The major difference being the expected better recall in the current experiment than in Cyr et al. because we used a shorter retention interval. Furthermore, using a repeated-measures design, this interaction has also been observed with an immediate serial recall task and an order reconstruction task (Kappel et al., 1973; Saint-Aubin et al., 2021).

The current findings clearly show that the interaction of the production effect with serial position is its signature and not a by-product of participant-controlled strategies. This finding has important implications for memory models accounting for the production effect. According to the relative distinctiveness view, in a within-list design, in which produced and silently read items are embedded within the same list, produced items are better recalled because they are more distinctive relative to silent items (e.g., Conway & Gathercole, 1990; MacLeod et al., 2010). With pure lists, produced items would lose their relative distinctiveness advantage and recall performance should be equivalent for both encoding conditions. The similar recall performance for produced and silent items observed here fits very well with the relative distinctiveness account. However, in its simplest form, the relative distinctiveness account cannot explain the large interaction between the production effect and serial positions. Furthermore, it has previously been noted that this view is incomplete because, among other things, it cannot predict the presence of a between-lists effect with an item recognition task and the pattern of costs and benefits of production when comparing performance between pure and mixed lists (see, e.g., Bodner et al., 2014; Fawcett, 2013; MacLeod & Bodner, 2017).

Fawcett (2013; Fawcett & Ozubko, 2016) suggested that a dual-process account in which relative distinctiveness cohabits with another process would provide the best explanation of the production effect. Memory strength and rehearsal have been proposed as potential candidates. However, none of the proposed architectures predict an interaction with serial positions. Recently, Saint-Aubin et al. (2021) presented a modified version of the Feature Model (Nairne, 1988, 1990) in which relative distinctiveness and rehearsal play key roles. This new architecture predicts the observed interaction between the production effect and serial positions.

### The Revised Feature Model

As its name implies, the RFM is an extension of the Feature Model (Nairne, 1988, 1990; Neath & Nairne, 1995; see also Poirier et al., 2019). In adapting the Feature Model, Saint-Aubin et al. (2021) retained the key elements, added a rehearsal component, and slightly modified the overwriting process. In broad strokes, within the RFM, the to-be-remembered items are represented by two types of features: modality-independent and modality-dependent features. Modality-independent features are generated by internal processes of categorization and identification. Modality-dependent features represent the physical presentation conditions, such as the color of the items or voice characteristics.

Within the RFM, item presentation simultaneously generates identical traces in primary and secondary memory. In both cases, items are represented by vectors of features, including modality-dependent and modality-independent features. In primary memory, vectors of features are degraded through similarity-based retroactive interference. That is to say that if a given feature of item n-1 is identical to the corresponding feature of item n, then this feature of item n-1 will be overwritten with some probability. Retroactive interference is not limited to the immediate previous item, but its strength decreases as the distance between items increases. After all list items have been presented, a final overwriting of modality-independent features only takes place due to continuing internal thought activity in preparation for recall.

Saint-Aubin et al. (2021; Cyr et al., 2021) implemented the production effect by assuming that produced items benefit from more modality-dependent features than silently read items. This is reminiscent of the implementation, within the original Feature Model, of the modality effect − that is the better recall of auditorily presented items compared to visually presented items on the last serial positions (Penney, 1989). Because, as mentioned above, the last item is followed by internally generated activity overwriting only modality-independent features, the last produced items would have more intact modality-dependent features. Therefore, produced items would be better recalled on the last serial positions.

Within the RFM, it is further assumed that overwritten features can be restored by a rehearsal process. More specifically, after the presentation of each list item, there is an attempt to rehearse all presented items so far. Some features are restored through this process. In accordance with empirical data, rehearsal efficiency drops as list length increases (see, e.g., Bhatarah et al., 2009; Rundus, 1971). Therefore, the first list items benefit more from the rehearsal process than the last ones. It is further assumed that producing an item by saying it aloud disrupts the rehearsal process in a way analogous to articulatory suppression (Murray, 1967; Saint-Aubin et al., 2021). Traces in secondary memory are assumed to remain intact. Consequently, on the first serial positions, silent items would be better recalled because they would benefit from more restored features than produced items.

As a final note, within the RFM, none of these processes are under participants’ control. Therefore, contrary to the compensating strategy hypothesis, the RFM predicts the same interaction between the production effect and serial position with a repeated-measures design and a between-participants design.

## Conclusion

The results from this study are clear and can be summarized as follows. When collapsed across serial positions, there was little to no difference in the recall performance between items being read aloud and items being read silently. However, as found in our systematic review of studies using a repeated-measures design, with a between-participants design, we observed a critical interaction between presentation modality and serial position. More specifically, recall performance for the first items presented in the list was better if those items were read silently than if they were read aloud, and the reverse pattern was found at the recency positions. The results suggest that producing the items increases their distinctiveness at the expense of hindering rehearsal.

## Footnotes

^1^
The same lists were used for all participants to provide greater control over list composition and to avoid introducing phonological or semantic similarity within a list as could occur with a random selection of words. Although this strategy can be suboptimal when the critical manipulation is at the item level, this is not the case here because the same lists were presented in both conditions. In fact, the conditions differ as a function of what participants are asked to do at encoding: They either read the items silently or they read them aloud. Therefore, the observed effects are unlikely to be a by-product of list composition.

## Notes

**Publication Ethics:** All participants gave their free and informed consent before the study, which was approved by the research ethics board of Université de Moncton.

**Open Data:** The stimuli are provided in the Appendix, and the data are available on the Open Science Framework project page https://osf.io/32msj (Gionet et al., 2022). Our article contains no experiment with a completely executed preregistration.

## Appendix

**Table A1:** Lists of stimuli used for practice and experimental trials in both presentation modality groups (read silently or read aloud)

Input serial position										
List	1	2	3	4	5	6	7	8	9	10
Practice 1	vie	Type	oeuvre	geste	croche	filtre	sens	teint	droite	trou
Practice 2	saut	Vitre	pont	touche	crise	fleur	clou	place	cygne	pire
Exp. 1	arbre	Pose	peste	veste	cidre	menthe	soeur	lourd	coupe	but
Exp. 2	plante	Contre	baisse	cape	fond	air	monde	somme	arche	puce
Exp. 3	cane	Lampe	foule	casque	tonne	boue	pied	poche	grotte	sexe
Exp. 4	cave	Roue	foie	eau	cendre	barre	linge	feuille	pipe	loutre
Exp. 5	poivre	Mousse	bond	note	haut	test	loge	rame	chute	pion
Exp. 6	blond	Proie	roche	porc	pli	jambe	miel	fraude	botte	grue
Exp. 7	site	Faim	jour	nouille	sueur	brave	lime	cuisse	mer	tigre
Exp. 8	douche	Plainte	maire	lait	rhume	couple	hanche	bave	centre	poule
Exp. 9	double	Jouet	ail	nuage	liste	plomb	tache	pain	belle	pou
Exp. 10	carte	Banc	code	tronc	rue	pelle	forme	pneu	vol	truite
Exp. 11	lac	Robe	boule	science	brin	norme	temple	flou	panne	gare
Exp. 12	sorte	Mime	scie	dieu	soie	coffre	vivre	chasse	ruse	fait
Exp. 13	Stade	Laine	crabe	louche	rire	route	chou	doigt	frappe	arme
Exp. 14	vache	Masque	crime	fable	muscle	glace	montre	butte	chose	dent
Exp. 15	Bord	Jupe	craie	face	poing	haute	perle	chiffre	hache	tuque
Exp. 16	bague	Nain	zone	sport	aile	hausse	cadre	heure	miette	colle
Exp. 17	Ruelle	Lisse	femme	coup	ongle	rythme	poudre	gorge	truc	huile
Exp. 18	Court	Cage	grippe	poil	bande	diable	ancre	tri	poste	lune
Exp. 19	Loupe	Bulle	vin	quille	grange	frite	oncle	gueule	tasse	cube
Exp. 20	Chef	Sang	luge	compte	oeil	graisse	angle	ombre	art	train
*Note*. Exp. = experimental lists.										

## References

[c1] Bhatarah, P., Ward, G., Smith, J., & Hayes, L. (2009). Examining the relationship between free recall and immediate serial recall: Similar patterns of rehearsal and similar effects of word length, presentation rate, and articulatory suppression. *Memory & Cognition*, *37*(5), 689–713. 10.3758/MC.37.5.689 19487760

[c2] Bodner, G. E., Taikh, A., & Fawcett, J. M. (2014). Assessing the costs and benefits of production in recognition. *Psychonomic Bulletin & Review*, *21*(1), 149–154. 10.3758/s13423-013-0485-1 23884689

[c3] Conway, M. A., & Gathercole, S. E. (1990). Writing and long-term memory: Evidence for a “translation” hypothesis. *The Quarterly Journal of Experimental Psychology Section A*, *42*(3), 513–527. 10.1080/14640749008401235

[c4] Covidence Systematic Review Software. Veritas Health Innovation Ltd., Melbourne, Australia. https://www.covidence.org

[c5] *Cyr, V., Poirier, M., Yearsley, J. M., Guitard, D., Harrigan, I., & Saint-Aubin, J. (2021). The production effect over the long term: Modeling distinctiveness using serial positions. *Journal of Experimental Psychology: Learning, Memory, and Cognition*. Advance online publication. 10.1037/xlm0001093 34726441

[c6] *Engle, R. W., Cantor, J., & Turner, M. L. (1989). Modality effects : Do they fall on deaf ears? *The Quarterly Journal of Experimental Psychology A: Human Experimental Psychology*, *41*(2-A), 273–292. 10.1080/14640748908402366 2748932

[c7] *Engle, R. W., & Roberts, J. S. (1982). How long does the modality effect persist? *Bulletin of the Psychonomic Society*, *19*(6), 343–346. 10.3758/BF03330277

[c8] Faul, F., Erdfelder, E., Lang, A.-G., & Buchner, A. (2007). G*Power 3 : A flexible statistical power analysis program for the social, behavioral, and biomedical sciences. *Behavior Research Methods*, *39*(2), 175–191. 10.3758/BF03193146 17695343

[c9] Fawcett, J. M., & Ozubko, J. D. (2016). Familiarity, but not recollection, supports the between-subject production effect in recognition memory. *Canadian Journal of Experimental Psychology/Revue canadienne de psychologie expérimentale*, *70*(2), 99–115. 10.1037/cep0000089 27244352 PMC4886847

[c10] Fawcett, J. M. (2013). The production effect benefits performance in between-subject designs: A meta-analysis. *Acta Psychologica*, *142*(1), 1–5. 10.1016/j.actpsy.2012.10.001 23142670

[c11] Forrin, N. D., & MacLeod, C. M. (2016). Order information is used to guide recall of long lists: Further evidence for the item-order account. *Canadian Journal of Experimental Psychology*, *70*(2), 125–138. 10.1037/cep0000088 27244354

[c12] Galli, G., Leng Choy, T., & Otten, L. J. (2012). Prestimulus brain activity predicts primacy in list learning. *Cognitive Neuroscience*, *3*(3–4), 160–167. 10.1080/17588928.2012.670105 22888370 PMC3413908

[c13] Gionet, S., Guitard, D., & Saint-Aubin, J. (2022). *Data for “The production effect interacts with serial positions: Further evidence from a between-subjects manipulation”*. https://osf.io/32msj 10.1027/1618-3169/a000540 PMC9446468 35272478

[c14] *Greene, R. L. (1985). Constraints on the long-term modality effect. *Journal of Memory and Language*, *24*(5), 526–541. 10.1016/0749-596X(85)90044-0

[c15] *Greene, R. L., & Crowder, R. G. (1986). Recency effects in delayed recall of mouthed stimuli. *Memory & Cognition*, *14*(4), 355–360. 10.3758/BF03202514 3762390

[c16] *Gregg, V. H., & Gardiner, J. M. (1984). Phonological similarity and enhanced auditory recency in longer-term free recall. *The Quarterly Journal of Experimental Psychology Section A*, *36*(1), 13–27. 10.1080/14640748408401501

[c17] *Grenfell-Essam, R., Ward, G., & Tan, L. (2017). Common modality effects in immediate free recall and immediate serial recall. *Journal of Experimental Psychology: Learning Memory and Cognition*, *43*(12), 1909–1933. 10.1037/xlm0000430 28557502 PMC5729966

[c18] Jones, A. C., & Pyc, M. A. (2014). The production effect: Costs and benefits in free recall. *Journal of Experimental Psychology: Learning, Memory, and cognition*, *40*(1), 300–305. 10.1037/a0033337 23751006

[c19] Kappel, S., Harford, M., Burns, V. D., & Anderson, N. S. (1973). Effects of vocalization on short-term memory for words. *Journal of Experimental Psychology*, *101*(2), 314–317. 10.1037/h0035247

[c20] Kass, R. E., & Raftery, A. E. (1995). Bayes factor. *Journal of the American Statistical Association*, *90*(430), 773–795. 10.1080/01621459.1995.10476572

[c21] Koriat, A. (2000). Control processes in remembering. In E. Tulving & F. I. M. Craik (Eds.), *The Oxford handbook of memory* (pp. 333–346). Oxford University Press.

[c22] Lambert, A. M., Bodner, G. E., & Taikh, A. (2016). The production effect in long-list recall: In no particular order? *Canadian Journal of Experimental Psychology*, *70*(2), 165–176. 10.1037/cep0000086 27244358

[c23] Lawrence, M. A. (2016). *Ez: Easy analysis and visualization of factorial experiments. R package version 4.4-0*. https://CRAN.R-project.org/package=ez

[c24] Macleod, C., Gopie, N., Hourihan, K., Neary, K., & Ozubko, J. (2010). The production effect : Delineation of a phenomenon. *Journal of Experimental Psychology: Learning, Memory, and Cognition*, *36*, 671–685. 10.1037/a0018785 20438265

[c25] MacLeod, C. M., & Bodner, G. E. (2017). The production effect in memory. *Current Directions in Psychological Science*, *26*(4), 390–395. 10.1177/0963721417691356

[c26] Morey, R. D. (2008). Confidence intervals from normalized data: A correction to Cousineau (2005). *Tutorial in Quantitative Methods for Psychology*, *4*(2), 61–64. 10.20982/tqmp.04.2.p061

[c27] Morey, R. D., & Rouder, J. N. (2018). *BayesFactor: Computation of Bayes Factors for common designs*. R package version 0.9.12-4.2. https://CRAN.R-project.org/package=BayesFactor

[c28] Morin, C., Poirier, M., Fortin, C., & Hulme, C. (2006). Word frequency and the mixed-list paradox in immediate and delayed serial recall. *Psychonomic Bulletin & Review*, *13*(4), 724–729. 10.3758/BF03193987 17201376

[c29] Murray, D. J. (1967). The role of speech responses in short-term memory. *Canadian Journal of Psychology*, *21*(3), 263–276. 10.1037/h0082978 6045500

[c30] *Murray, D. J., Leung, C., & McVie, D. F. (1974). Vocalization, primary memory and secondary memory. *British Journal of Psychology*, *65*(3), 403–413. 10.1111/j.2044-8295.1974.tb01414.x

[c31] Nairne, J. (1990). A feature model of immediate memory. *Memory & cognition*, *18*, 251–269. 10.3758/BF03213879 2192233

[c32] Nairne, J. S. (1988). The mnemonic value of perceptual identification. *Journal of Experimental Psychology: Learning, Memory, and Cognition*, *14*(2), 248–255. 10.1037/0278-7393.14.2.248 2856679

[c33] Navarro, D. J. (2015). *Learning statistics with R: A tutorial for psychology students and other beginners* (Version 0.5). University of Adelaide.

[c34] Neath, I., & Nairne, J. S. (1995). Word-length effects in immediate memory: Overwriting trace decay theory. *Psychonomic Bulletin & Review*, *2*(4), 429–441. 10.3758/BF03210981 24203783

[c35] New, B., Pallier, C., Brysbaert, M., & Ferrand, L. (2004). Lexique 2 : A new French lexical database. *Behavior Research Methods, Instruments, & Computers*, *36*(3), 516–524. 10.3758/BF03195598 15641440

[c36] Oberauer, K., Lewandowsky, S., Awh, E., Brown, G. D. A., Conway, A., Cowan, N., Donkin, C., Farrell, S., Hitch, G. J., Hurlstone, M. J., Ma, W. J., Morey, C. C., Nee, D. E., Schweppe, J., Vergauwe, E., & Ward, G. (2018). Benchmarks for models of short-term and working memory. *Psychological Bulletin*, *144*(9), 885–958. 10.1037/bul0000153 30148379

[c37] Parker, J. F. (1981). The locus of imagery effects in free recall. *The American Journal of Psychology*, *94*(1), 113–124. 10.2307/1422346

[c38] Penney, C. G. (1989). Modality effects and the structure of short-term verbal memory. *Memory & Cognition*, *17*, 398–422. 10.3758/BF03202613 2668697

[c39] Poirier, M., Yearsley, J. M., Saint-Aubin, J., Fortin, C., Gallant, G., & Guitard, D. (2019). Dissociating visuo-spatial and verbal working memory: It’s all in the features. *Memory & Cognition*, *47*(4), 603–618. 10.3758/s13421-018-0882-9 30560471 PMC6517348

[c40] Rohatgi, A. (2020). *WebPlotDigitizer* (Version 4.5) [computer software]. https://automeris.io/WebPlotDigitizer

[c41] Rouder, J. N., Morey, R. D., Speckman, P. L., & Province, J. M. (2012). Default Bayes factors for ANOVA designs. *Journal of Mathematical Psychology*, *56*(1), 356–374. 10.1016/j.jmp.2012.08.001

[c42] Rouder, J. N., Speckman, P. L., Sun, D., Morey, R. D., & Iverson, G. (2009). Bayesian t tests for accepting and rejecting the null hypothesis. *Psychonomic Bulletin & Review*, *16*(2), 225–237. 10.3758/PBR.16.2.225 19293088

[c43] Rundus, D. (1971). Analysis of rehearsal processes in free recall. *Journal of Experimental Psychology*, *89*(1), 63–77. 10.1037/h0031185

[c44] Saint-Aubin, J., Yearsley, J. M., Poirier, M., Cyr, V., & Guitard, D. (2021). A model of the production effect over the short-term: The cost of relative distinctiveness. *Journal of Memory and Language*, *118*, 104219. 10.1016/j.jml.2021.104219

[c45] Stoet, G. (2017). PsyToolKit: A novel web-based method for running online questionnaires and reaction-time experiments. *Teaching of Psychology*, *44*, 24–31. 10.1177/0098628316677643

[c46] Stoet, G. (2010). PsyToolkit: A software package for programming psychological experiments using Linux. *Behavior Research Methods*, *42*, 1096–1104. 10.3758/BRM.42.4.1096 21139177

[c47] Unsworth, N., Brewer, G. A., & Spillers, G. J. (2011). Inter- and intra-individual variation in immediate free recall: An examination of serial position functions and recall initiation strategies. *Memory*, *19*(1), 67–82. 10.1080/09658211.2010.535658 21240749

[c48] Watkins, M. J., LeCompte, D. C., & Kim, K. (2000). Role of study strategy in recall of mixed lists of common and rare words. *Journal of Experimental Psychology*, *26*(1), 239–245. 10.1037/0278-7393.26.1.239 10682300

[c49] *Watkins, M. J., Watkins, O. C., & Crowder, R. G. (1974). The modality effect in free and serial recall as a function of phonological similarity. *Journal of Verbal Learning & Verbal Behavior*, *13*(4), 430–447. 10.1016/S0022-5371(74)80021-6

